# Undergraduate radiology education in Europe in 2022: a survey from the European Society of Radiology (ESR)

**DOI:** 10.1186/s13244-023-01388-8

**Published:** 2023-02-24

**Authors:** European Society of Radiology (ESR), Francisco Sendra-Portero, Miguel Souto, Minerva Becker, Vicky Goh

**Affiliations:** European Society of Radiology (ESR), Am Gestade 1, 1010 Vienna, Austria

**Keywords:** Schools (medical), Curriculum, Radiology, Surveys and Questionnaires, Diagnostic Imaging

## Abstract

**Background:**

Education in radiology should be an integral aspect of undergraduate medical training given the essential role of imaging in patient management. Since the introduction of the European Society of Radiology undergraduate curriculum a decade ago, radiology education has evolved.

**Objectives:**

This survey aimed to assess the current status of undergraduate radiology education in Europe.

**Methods:**

An electronic survey on undergraduate teaching was distributed by the European Society of Radiology to delegates of the European Society of Radiology education committee and presidents of national radiological societies from April 1 to May 31, 2022. Data from the twenty questions were summarized using descriptive statistics.

**Results:**

There were 72 respondents from 36 countries. Radiology was taught to undergraduates in 95% (68/72), with a national or local curriculum informing radiology education in 93% (67/72). Radiology teaching was delivered by radiologists in 98% (58/59), across all years of medical school but most commonly in the fourth year of medical training (63%, 44/70), through various means including lectures, workshops, radiology department placements, online resources and simulation. Teaching hours were variable, with a minimum of 10 h reported.

**Conclusion:**

This survey’s findings suggest an improvement over the last decade in the engagement of radiologists in the delivery of undergraduate radiology education in European countries affiliated with the European Society of Radiology.

**Supplementary Information:**

The online version contains supplementary material available at 10.1186/s13244-023-01388-8.

## Key points

1. Survey findings indicate improvements have been made in the last decade in radiologists’ involvement in undergraduate radiology education.
2. Undergraduate radiology education is curriculum-based in over 90% of ESR-affiliated European countries surveyed.
3. Teaching is now delivered by radiologists across all years of medical training through lectures, workshops, radiology department placements, online resources and simulation.

**Supplementary Information:**

The online version contains supplementary material available at 10.1186/s13244-023-01388-8.

## Introduction

Medical imaging plays a significant role in patient management, and radiology is an important aspect of undergraduate medical training [1]. The involvement of radiologists in undergraduate teaching is fundamental to ensure that the doctors of tomorrow are equipped with relevant up-to-date knowledge to practice safely; that they are able to order the appropriate imaging tests for their patients; and that working together with the radiologist, they will be able to action management appropriately depending on the results.

Harmonization of undergraduate education across Europe has been a key component to ensuring equality. The Bologna process sought to standardize higher education across Europe [2, 3], and the European Society of Radiology (ESR) undergraduate curriculum [4] was introduced over a decade ago to provide a basis for radiology curricula in medical schools across Europe aiming to address ongoing variation across different countries in undergraduate education in radiology [5].

The ESR undergraduate curriculum is a working document and has been updated every three to four years since its introduction in 2011 [6]. In recognition of ongoing transformations to undergraduate teaching, this ESR survey was undertaken to determine current status of undergraduate radiology education in European medical schools, to inform future iterations of the ESR undergraduate curriculum and to highlight methods to improve engagement with medical students in radiology.

## Methods

An online survey was conducted from April 1 to May 31, 2022, by the ESR of members of the Education Committee and national society delegates. The survey consisted of 20 questions related to the status of undergraduate radiology teaching in the respondents’ respective countries (Additional file 1). Broadly, these covered when and how radiology was taught; knowledge and utilization of the ESR curriculum; and included how engagement of medical students in radiology could be improved. Survey results were summarized with descriptive statistics.

## Results

Seventy-two respondents from 36 countries responded to the survey, which represents 73% of the European countries affiliated to the ESR. There were no data from the following 13 countries: Albania, Bosnia and Herzegovina, France, Georgia, Israel, Kyrgyzstan, Luxembourg, Moldova, North Macedonia, Poland, Portugal, Serbia, Uzbekistan. There were varying response rates depending on the question posed (median 84%, range: 0–100%).

The number of medical schools in the 36 respective countries varied (median 7, range 1–120). The number of radiology professors affiliated with a university was reported to be more than 10 in number by 43% of respondents and more than 20 by 31% of respondents. Sixty-five percent (47/72) of respondents indicated that there was a national curriculum in their country for radiology. For the respondents reporting the lack of a national radiology curriculum, 80% (20/25) reported using a local curriculum (set by the university, medical school and/or hospital radiology department); 4% (1/25) the ESR undergraduate curriculum; and 16% (4/25) no curriculum at all.

**Fig. 1:**
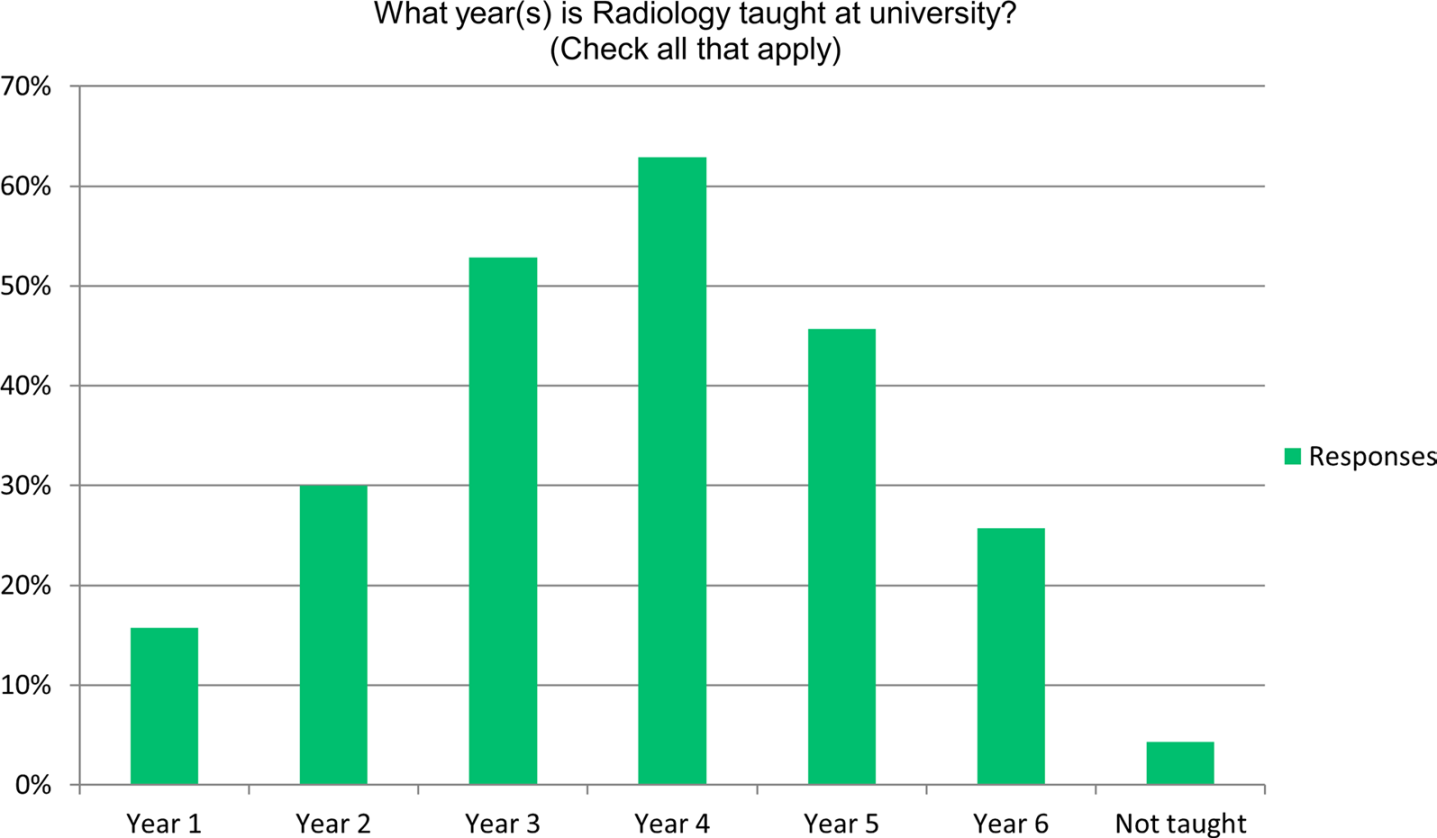
Frequency plot showing distribution of radiology teaching across the year of medical school

In terms of when and how radiology education was delivered, radiology was taught across the breadth of medical school education (Fig. 1), with 16% (11/72) reporting radiology was introduced in year 1 of medical school, increasing in the later years (3–6 years) and peaking at year 4 of medical school, with 63% (44/70) of respondents, though in 4% (3/70) of respondents, radiology was not taught at all. The hours reportedly dedicated to radiology teaching varied tremendously across institutions within a country and between countries, median 64.5 h, range 10–500 h.

**Fig. 2:**
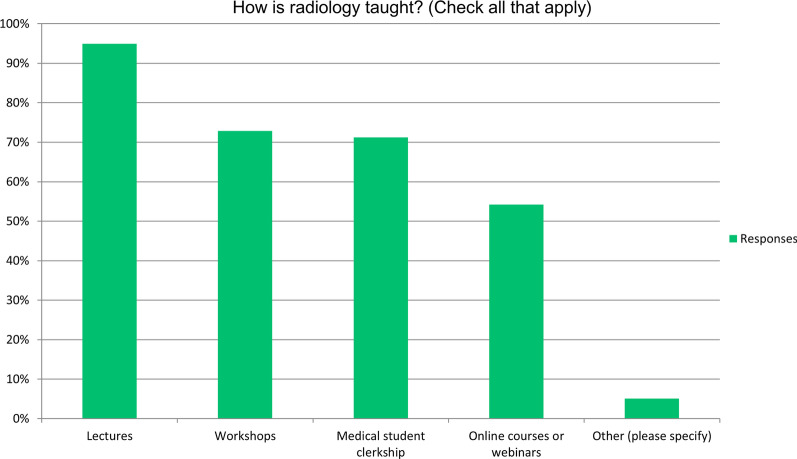
Frequency plot showing distribution of teaching formats used to deliver radiology education

Radiology was taught as a standalone subject in the majority of institutions (54%, 32/59). In 31% (18/59), radiology teaching was integrated within other subjects, e.g., internal medicine or surgery; or a combination of standalone and integrated teaching (15%, 9/59). This was through a number of different formats including lectures, workshops and practical experience through medical student clerkships, and included both face-to-face and online teaching through online courses or webinars (Fig. 2). A few (5%, 3/59) of respondents also reported the use of other innovations such as simulation.

**Fig. 3:**
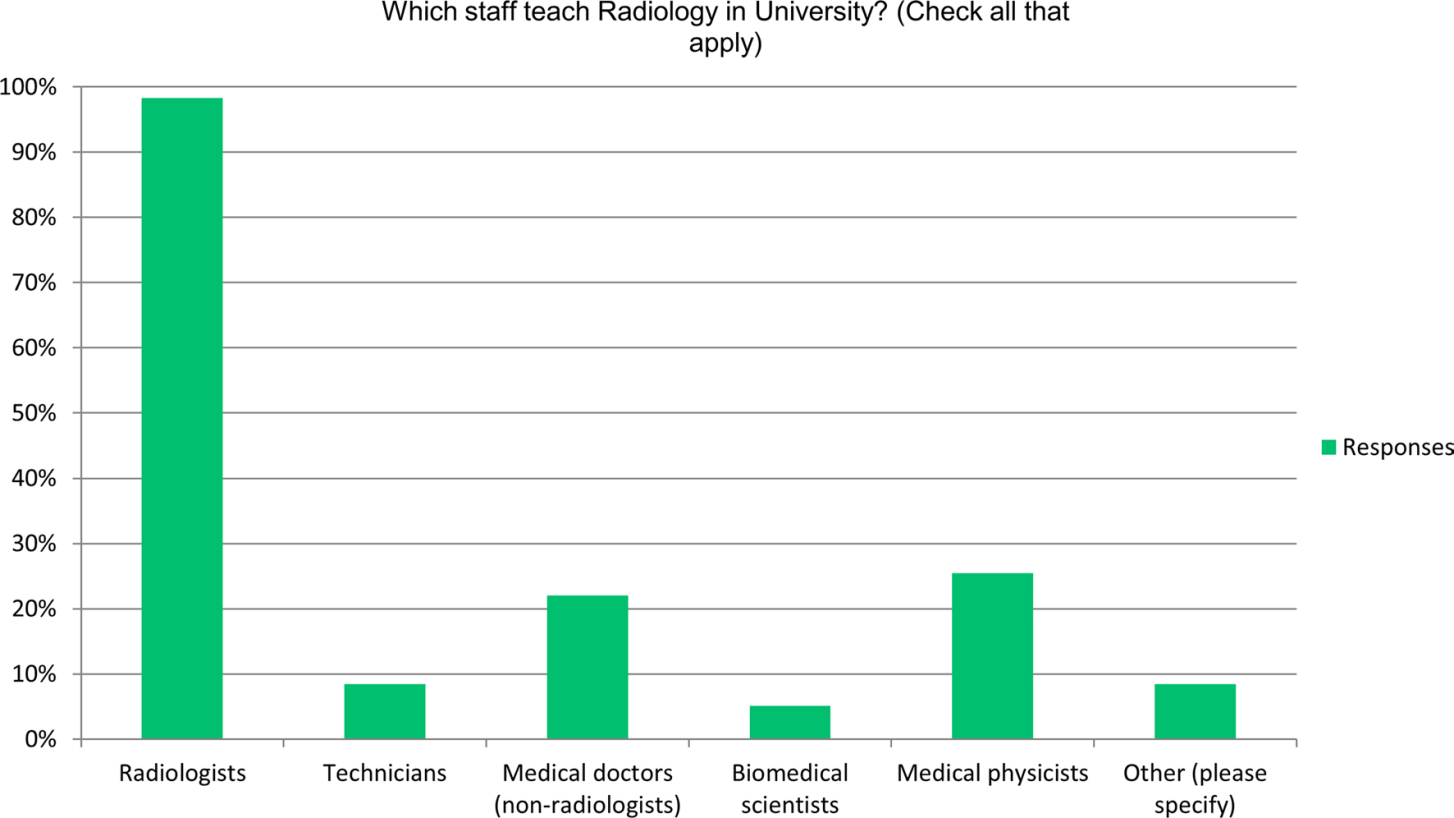
Frequency plot showing the type of staff delivering radiology education

Teaching was delivered by radiologists in 98% (58/59), with other medical doctors and medical physicists/biomedical scientist contributing in 22% (13/59) and 30% (18/59), respectively (Fig. 3).

Medical schools also offered opportunity for more in-depth study to medical students, including research projects dedicated to radiology in 60% (37/62), and in 37% (23/62), there were additional degrees dedicated to medical imaging at Bachelor (BSc), Master (MSc) or Doctorate (PhD) levels.

In terms of the ESR undergraduate curriculum, 79% (49/62) were aware of its existence. While a national curriculum was in the place for the majority of respondents, 67% (33/49) respondents answered that the ESR undergraduate curriculum components parts were included in undergraduate teaching.

In terms of the decision toward radiology as a specialty, this was made in year 5 of medical school and beyond in the majority of cases (87%, 54/62). In general, respondents indicated that there should be greater practical experience available to medical students, greater visibility of radiology and greater engagement of students by radiologists to attract young doctors into radiology. Of note 31% (19/62) reported the presence of undergraduate radiological societies contributing to the visibility and value of radiology as a clinical specialty.

## Discussion

In the decade since the introduction of the European Society of Radiology (ESR) undergraduate curriculum, undergraduate radiology education has evolved with the modernization of medical curricula as well as teaching practices [7] and ongoing efforts at harmonization. The current ESR survey follows previous ESR surveys (published and unpublished) and has highlighted the following. With the exception of a small minority of countries where there appears to be a lack of dedicated undergraduate radiology teaching (< 5% of respondents), radiology is an established part of undergraduate medical education, across all years of medical school. In total, 93% of respondents indicated that radiology education is based on a curriculum, which is essential to delivery of quality education, and is an improvement from a decade ago. In total, 75% of respondents also highlighted this was a national curriculum, which is higher than the 50% of a prior unpublished ESR survey in 2020.

There is greater involvement of radiologists in delivering undergraduate teaching compared to a decade ago, with 98% reporting that this is delivered by radiologists, alongside clinicians and scientists. This is important for students in terms of deeper insights from radiology, and for the clinical speciality of radiology, greater visibility. There is also diversity in teaching methods including the incorporation of online active and blended learning approaches and simulations, in part propelled by the need for alternative means of delivering education during the COVID pandemic [8]. Simulations involve a variety of patient model and computer-based programs mimicking real-world situations. Virtual reality provides an immersive experience, e.g., of anatomical dissection and interventional procedures.

These evolving teaching approaches have been shown to improve medical students' performance, satisfaction and engagement [9]. There also remains a strong trend for placements in radiology departments, and an opportunity to undertake research projects, and even research degrees, reported by 60% and 37% of respondents, respectively, which provides more in-depth exposure to advances in radiology. In particular, the opportunity to undertake a research degree has increased from 17% in the prior unpublished 2020 ESR survey, highlighting the efforts of university-affiliated radiologists to improve the prospect of medical students becoming interested in clinical radiology as a career specialty.

The survey also highlighted there is still heterogeneity in terms of the delivery of radiology teaching, and teaching hours (or number of credits) dedicated to radiology, ranging from 10 to 500 h in this survey reflecting ongoing differences in curricula type (conventional, problem based or hybrid medical curricula). However, even at the upper limit of the range of teaching hours, this is well below 5% of total available undergraduate teaching hours. There remains a paucity of data on the optimal number of teaching hours in order to develop sufficient radiological competencies, but more work is needed by the ESR education committee, toward defining minimal requirements and benchmarking of teaching hours. Even in European countries with well-established national radiological curricula, studies have shown that radiology teaching hours only represent a small fraction of the undergraduate teaching [10]. Previous studies have highlighted lack of time within the curriculum and resistance from other departments as barriers for change [11]. Further progress is also needed into provision of frameworks for delivery of radiological education given new learning tools [8]. For example, Collaborative Online International Learning (COIL) is an approach that may provide opportunities globally for intercultural radiology learning.

In terms of attracting medical students into radiology, in this survey 54% of radiology is still taught as a standalone subject. While this reflects conventional medical school curricula, facilitates content management and evaluation of competencies from the educator’s perspective and provides an overview of radiology to medical students, it has long been recognized that an integrated approach to radiology education is more effective [12]; students are more motivated and engaged when direct clinical utility is evident [13, 14]. Earlier exposure of medical students to clinical radiology improves their understanding of the imaging tests that they will order as young doctors, as well as communication with patients and their patient’s experience [15]. Early exposure may well increase their interest in undertaking a radiology elective, undertaking radiology research and eventually specializing in radiology [16]; though one study has found no association between teaching hours in radiology and medical student career choices [17].

One might question the usefulness of the ESR undergraduate curriculum given the use of national and local curricula in the majority of respondents. However, it is important for the ESR to provide a template upon which national and local curricula can be based, and it is reassuring that components of the curriculum are implemented in respondents’ respective institutions or countries (increasing from 58% in the previous unpublished 2020 survey to 67% currently), and provides a framework for radiologists undertaking undergraduate teaching. For countries where there is no such process, the ESR curriculum remains a valuable resource. On the other end of the spectrum, some respondents indicated that the ESR curriculum was too comprehensive for undergraduate teaching, hence why this was not implemented in their institution/country. The ESR undergraduate curriculum was intended to be inclusive with core as well as more advanced aspects that would be more suited for students with a special interest in radiology to provide a continuum for learning.

There are a number of limitations to this survey. First, the survey was completed by ESR members rather than individual European medical schools, which will present a selection bias. Future surveys should address this bias. Second, not all European countries were represented in the survey including lack of respondents from France, Portugal and Poland. Third, response bias is typical in survey-based studies. Data were not obtained from a stratified sample that considered specific institutional or country characteristics, and the reliability of certain responses may be questioned. However, the distribution of the survey was supervised by ESR and included membership of the education committee and national societies. Fourth, this survey has only included a limited number of questions to ensure a good response rate and comparability to prior surveys. Some aspects of radiology teaching may not have been captured through free text options were available to respondents to provide further comments. Evaluation was also not a focus of the current survey.

In conclusion, over the last decade there have been greater engagements of radiologists in the delivery of radiology education within medical schools in European countries affiliated to the ESR. Despite efforts toward harmonization, there is still ongoing variation within and across countries in education delivery. Moving forward, updated ESR guidance on minimum teaching hours for radiological competency, a framework for effective delivery of teaching that includes novel methods, and more outcome data are needed for future benchmarking.

## Supplementary Information

**Additional file 1.** Survey questionnaire.

## Data Availability

Not applicable.

## Abbreviations

BSc: Bachelor of Science
ESR: European Society of Radiology
ESR: European Society of Radiology
MSC: Master of Science
PhD: Doctor of Philosophy
