# Supplementary material for: Undergraduate radiology education in Europe in 2022: a survey from the European Society of Radiology (ESR)
Source: Insights Imaging. 2023 Feb 24;14:37. doi: 10.1186/s13244-023-01388-8 (PMC9958208; doi:10.1186/s13244-023-01388-8)
Supplement: Supplementary file 1 — Additional file 1. Survey questionnaire. [file 13244_2023_1388_MOESM1_ESM.pdf]

ELECTRONIC SUPPLEMENTARY MATERIAL

Undergraduate Radiology Education in Europe in 2022: A survey from the European Society of Radiology (ESR)

Appendix 1: Survey questionnaire

1. **Where are you from?**

2. **How many universities with medical schools are there in your country?**

3. **How many university-affiliated radiology professors are there in your country?**
   0 / 1-5 / 6-10 / 11-15 / 16-20 / More than 20 / I don’t know

4. **Is there a national curriculum for radiology?**
   Yes / No

5. **Which curriculum is used instead?**
   There is no curriculum / ESR Curriculum used / Local curriculum used

6. **Who sets this local curriculum?**
   University / Medical School / Hospital Radiology Department / Other (please specify)

7. **What year(s) is Radiology taught at university? (Check all that apply)**
   Year 1 / Year 2 / Year 3 / Year 4 / Year 5 / Year 6 / Not taught

8. **How many pre-graduate hours dedicated to radiology do medical students have during their entire university curriculum?**

9. **Is radiology taught:**
   As a stand-alone subject / Integrated with subjects from other medical specialities e.g. internal medicine, cardiology, surgery /Other (please specify)

10. **How is radiology taught? (Check all that apply)**
    Lectures / Workshops / Placements in radiology departments / Online courses or webinars / Other (please specify)

11. **Which staff teach Radiology in University? (Check all that apply)**
    Radiologists / Technicians / Medical doctors (non-radiologists) / Biomedical scientists / Medical physicists / Other (please specify)

12. **Is there a specialist undergraduate degree dedicated to Radiology e.g. BSc, MSc, PhD?**
    Yes / No

13. **Do medical schools in your country offer research projects dedicated to radiology separate from a specialist undergraduate degree?**
    Yes / No

14. **Are there any undergraduate radiological societies in your country?**
    Yes / No

15. **In general, at what point during their medical education do students in your country decide to become radiologists?**
    Year 1 / Year 2 / Year 3 / Year 4 / Year 5 / Year 6 / Other (please specify)

16. **Are you aware of the ESR undergraduate curriculum?**
    Yes / No

17. **The content of the ESR Undergraduate Curriculum is:**
    Just the right level / Not comprehensive enough / Too comprehensive

18. **Is the ESR Undergraduate Curriculum or aspects of the ESR Curriculum implemented in undergraduate teaching in your country?**
    Yes / No

19. **Is there a reason why the ESR undergraduate curriculum is not implemented?**

20. **What could be improved to increase engagement of medical students in Radiology?**
